# Impact of Polysorbate 80 on the Antimicrobial Activity of Oregano and Thyme

**DOI:** 10.3390/molecules30010081

**Published:** 2024-12-28

**Authors:** Marta Carvalho, Joana Barbosa, Marcelo Belchior Rosendo da Silva, Helena Albano, Paula Teixeira

**Affiliations:** 1 Universidade Católica Portuguesa, CBQF-Centro de Biotecnologia e Química Fina—Laboratório Associado, Escola Superior de Biotecnologia, Rua de Diogo Botelho 1327, 4169-005 Porto, Portugal; s-mipcarvalho@ucp.pt (M.C.); jbarbosa@ucp.pt (J.B.); marcelo.brs@outlook.com (M.B.R.d.S.); 2 Escola Superior de Enfermagem de Coimbra, 3004-011 Coimbra, Portugal; microhel@gmail.com; 3 CISAS—Center for Research and Development in Agrifood Systems and Sustainability, Instituto Politécnico de Viana do Castelo, Rua Escola Industrial e Comercial de Nun’Álvares, 4900-347 Viana do Castelo, Portugal; 4 Escola Superior Agrária, Instituto Politécnico de Viana do Castelo, R. D. Mendo Afonso 147, Refóios, 4990-706 Ponte de Lima, Portugal

**Keywords:** foodborne pathogens, surfactant, plant volatiles, antimicrobial agents

## Abstract

Plant-derived essential oils (EOs) possess significant antimicrobial potential against spoilage and pathogenic microorganisms. However, their efficacy can vary depending on the test method, making it difficult to standardise results. This study aimed to investigate the effect of polysorbate 80, a common surfactant used to emulsify EOs, on antimicrobial activity and minimum inhibitory concentration (MIC) determinations. The antimicrobial activity of oregano and thyme EOs was tested against 40 microorganisms with and without the presence of polysorbate 80. Antimicrobial activity was qualitatively assessed using the disc diffusion assay (DDA) and quantitatively via broth microdilution to determine MIC values. Both oregano and thyme EOs exhibited antimicrobial activity against all tested microorganisms in the DDA, regardless of the surfactant’s presence. However, MIC determinations revealed that higher EO concentrations were required to inhibit microbial growth when polysorbate 80 was included in the emulsification process. These findings indicate that polysorbate 80 influences antimicrobial test results by reducing EO efficacy while enhancing solution homogeneity and handling in aqueous media. The study highlights the critical role of emulsifiers in antimicrobial testing, as their use can significantly impact the interpretation of results and the perceived effectiveness of EOs in food preservation, pharmaceuticals, and other applications.

## 1. Introduction

The rise of antibiotic-resistant bacteria is one of the most critical public health threats globally. Pathogenic bacteria have developed a variety of resistance mechanisms, leading to a dramatic reduction in the effectiveness of many traditional antibiotics, which has serious consequences for the treatment of infectious diseases. The growing resistance crisis has driven researchers to explore alternative antimicrobial agents, including essential oils (EOs) derived from aromatic and medicinal plants, as potential solutions [1,2,3,4]. The hydrophobic components of EOs insert themselves into lipid membranes, separating lipids from bacterial cell membranes and mitochondria. This disruption results in alterations to membrane permeability and/or ATP generation [5,6]. Despite this potential, several challenges hinder the practical application of EOs as antimicrobial agents.

Oregano (*Origanum vulgare*) and thyme (*Thymus vulgaris*) EOs have been extensively studied for their antimicrobial properties, owing to their rich content of bioactive compounds, particularly phenolic derivatives such as carvacrol and thymol. These compounds exhibit strong antibacterial and antifungal activities, making oregano and thyme EOs attractive candidates for addressing the growing threat of antibiotic-resistant pathogens [7,8]. For example, their efficacy against multidrug-resistant bacteria such as *Staphylococcus aureus* (including MRSA) and *Bacillus cereus* has been proven [9,10]. Their ability to inhibit the formation of biofilms—a major factor in persistent infections—further highlights their potential in clinical applications. Moreover, these EOs are widely recognized for their antioxidant properties, which can play a supportive role in mitigating oxidative stress during infections [9,10].

Essential oils are volatile, water-insoluble, and viscous, properties that make them difficult to work with in standard laboratory antimicrobial assays. These characteristics complicate their dilution and lead to uneven distribution within the testing medium [11]. As a result, inconsistent results are often reported across studies, raising questions about the reliability of EO-based antimicrobial testing [12,13,14]. This variability makes it difficult to compare t of results between studies and limits the potential for standardization, which is essential for developing EO-based products for clinical or industrial applications. A variety of methods are used to assess the antimicrobial activity of EOs, falling broadly into qualitative and quantitative categories. Qualitative methods, such as disc diffusion and well diffusion assays, are widely used because of their simplicity and speed. Quantitative methods, like broth macro/microdilution and agar dilution, are more rigorous and provide precise measurements of antimicrobial efficacy [12,13,15]. However, diffusion methods have been criticized for their unreliability, particularly with EOs, due to issues with volatility and oil distribution, which can lead to false results or underestimation of antimicrobial potential [15,16]. Another critical challenge is achieving a homogeneous mixture of EOs in the test medium. Essential oils do not dissolve easily in water, making it difficult to achieve consistent results without the use of surfactants. Common surfactants such as polysorbate 80, dimethylsulfoxide (DMSO), and ethanol help solubilize and disperse EOs more evenly in aqueous solutions [17,18,19]. Among these, polysorbate 80, a non-ionic surfactant, is frequently used in antimicrobial assays due to its ability to emulsify EOs without interfering with their antimicrobial activity. In the study of Hilbig et al. [20], no inhibition was observed for three bacterial species after polysorbate 80 treatments up to 60,000 ppm. It improves EO miscibility in the test medium and enhances the stability of the emulsion, which is crucial for ensuring the reproducibility and reliability of test results.

The objectives of this study were (i) to evaluate the antimicrobial activity of oregano and thyme EOs emulsified with polysorbate 80 against 40 microorganisms by disc diffusion assay and to determine the minimum inhibitory concentrations by microtitre plate dilution and (ii) to compare the results with those obtained for the same EOs and isolates without the surfactant polysorbate 80. These EOs were selected as they were those demonstrating the highest inhibitory activity, with the lowest MIC values, in a preliminary screening including 23 EOs [21].

## 2. Results

### 2.1. Antimicrobial Activity

**Figure 1:**
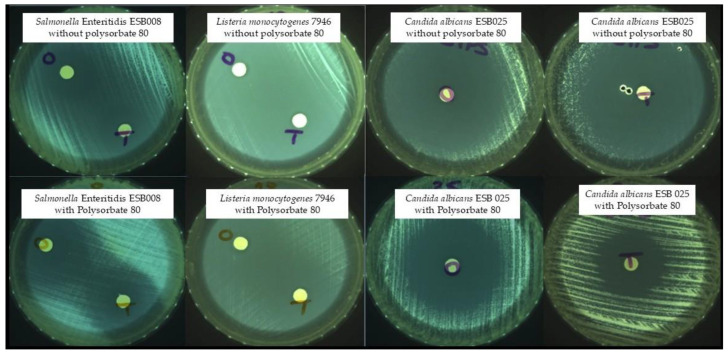
Zones of growth inhibition—O: oregano; T: thyme.

The most common method for screening the in vitro antimicrobial activity of plant EOs is to measure the diameter of the inhibition zone of bacterial growth on agar by disc diffusion assay. Some examples of the obtained inhibition zones are shown in Figure 1.

Table 1 shows the inhibition zone diameters (mm) of each EO with and without polysorbate 80 against the 40 microorganisms tested. These results were previously presented at the national MICROBIOTEC’21—Microbiology and Biotechnology Congress [21]. Both EOs, with and without polysorbate 80, showed activity against all the microorganisms tested (Table 1). *Clostridium sporogenes* (1.31, 1.34 and 1.61), *Clostridium perfringens* (1.16 and 1.19), *Listeria monocytogenes* SCOTT A, *Klebsiella pneumoniae* ESB011, and *Salmonella* Enteritidis (ESB008, 417536, and 545047) proved to be the most resistant to both EOs.

### 2.2. Determination of Minimum Inhibitory Concentration (MIC)

In determining the MICs, it was observed that the results obtained with and without surfactant were markedly different (Table 2, [21]). These results are in agreement with those obtained in the disc diffusion test since the microorganisms identified as the most resistant (*C. sporogenes* (1.31, 1.34, and 1.61), *C. perfringens* (1.16 and 1.19), *L. monocytogenes* SCOTT A, *K. pneumoniae* ESB011, and *Salmonella* Enteritidis ESB008) showed high MICs (between 3.12 and 6.25%). The results for oregano EO with polysorbate 80 showed MIC values between 0.39 and 12.50%, while the same EO without polysorbate showed MICs between 0.02 and 0.39%. Similarly, thyme EO with polysorbate 80 had MICs between 0.78 and 12.50% and without polysorbate between 0.02 and 0.39%.

## 3. Discussion

Essential oils can have bacteriostatic (inhibiting) or bactericidal (killing) effects [22]. Comparing the results obtained in this experiment for DDA, in the study carried out by Carvalho et al. [23] for oregano and thyme EO without surfactant, similar results were obtained, with inhibition zones varying between 22 and 76 mm for all the microorganisms tested (the same used in this study), indicating that this methodology is reproducible. Similar results were obtained by Dorman and Deans [24] for thyme EO, and Mith et al. [25] and Puškárová et al. [26] showed that for oregano EO without surfactant, inhibition zones between 25 and 55 mm were obtained for the studied strains of *Salmonella* Typhimurium and *L. monocytogenes*. Comparing the two procedures performed, significant differences were observed only for the thyme EO (*p* < 0.05). However, although few studies were performed with a surfactant, studies performed with DMSO showed small inhibition halos for *E. coli*, *Salmonella* Typhimurium, *S. aureus*, *C. albicans,* and *C. perfringens* [27,28], contrary to our study. No studies were found on the use of polysorbate 80 in the disc diffusion test.

For MICs, the results obtained in this experiment with oregano EO without surfactant were similar to those of the study by Carvalho et al. [23], which showed MIC values between 0.1950 and 0.0244%. Other authors confirmed that lower MICs were found for *E. coli*, *S. aureus*, *L. monocytogenes*, *E. faecalis*, *Salmonella* Typhimurium, *Salmonella* Enteritidis, *Y*. *enterocolitica,* and *B. cereus*, using the same method [10,26,29]. For thyme EO, Friedman et al. [29] showed that *E. coli*, *Salmonella* Enteritidis, and *L. monocytogenes* showed results consistent with this experiment, and Radaelli et al. [30] showed lower MIC values for *C. perfringens*. However, other similar studies have shown higher MICs for *C. perfringens* than those found in this study [31,32,33]. Different inhibitions for the same microorganisms can be explained by different chemical compositions of the EOs, which vary according to the season, the geographical location of the plants, and/or the method used to extract the EOs, as well as the microbial strains used in the assays [34,35]. However, when surfactants are used, several authors have shown that with the use of Tween 80 or DMSO, the MICs were lower than those determined in this study for several microorganisms [9,27,28]. Remmal et al. [36] found that the antibacterial activity of EOs decreased when polysorbate was used. Although there is no consensus in the studies on EOs and the use of surfactants, these compounds appear to be important as polysorbate 80 is used to emulsify the oil, making it miscible with the medium used in the dilutions of the wells [17,37]. The use of emulsifiers/solvents could limit or enhance the antimicrobial activity of EOs, and, to date, there are many studies with controversial results. However, as a surfactant, it appears that polysorbate 80 possesses several beneficial properties that enhance the application of essential oils (EOs). By reducing the surface tension between the oil and the aqueous phase, polysorbate 80 enables the oils to become miscible with the surrounding solution, resulting in a more uniform distribution of the EOs. This enhanced homogenization is crucial for ensuring consistent dosing and efficacy across different applications [38]. Moreover, using polysorbate 80 can help stabilize emulsions of EOs, preventing phase separation and ensuring that the antimicrobial compounds remain evenly distributed over time. This stability is particularly important in formulations intended for commercial use, where consistent performance is critical [39]. Despite the potential drawbacks of reduced antimicrobial activity when EOs are used with polysorbate 80, these surfactant properties cannot be overlooked. As Vel et al. [40] suggested, the surfactant’s ability to improve the miscibility of oils and enhance cell membrane permeability highlights its significance in formulating effective antimicrobial treatments. A possible explanation for these differences may be related to the distribution of the essential oil in the 96-well plates. In the absence of polysorbate 80, two distinct phases are formed, potentially exposing microorganisms to higher localized concentrations of EO. Conversely, polysorbate allows the homogenization of the oil in the medium. This homogeneous distribution allows bacterial growth at what appears to be higher overall concentrations but may actually represent lower localized exposure to the active compounds in the EO. Therefore, while further research is needed to fully understand the balance between the surfactant’s benefits and its impact on EO efficacy, polysorbate 80 remains a valuable component in the development of formulations that harness the antimicrobial properties of essential oils [41].

## 4. Materials and Methods

### 4.1. Essential Oils and Microorganisms Tested in This Study

Two essential oils supplied by Infinite Choice (Coimbra, Portugal) were used in this study: oregano (*Origanum vulgare* leaf) and thyme (*Thymus vulgare* leaf). All the strains used in this study (Table 3) were stored at −20 °C in Tryptic Soy broth with 6 g/L of Yeast Extract (TSBYE; Biokar Diagnostics, Allonne, France) containing 30% (*v*/*v*) glycerol (Sigma, Steinheim, Germany), and subcultured twice before use in the assays. Each bacterial strain was grown on Tryptic Soy Agar with 6 g/L of Yeast Extract (TSAYE, Biokar Diagnostics) at 37 °C for 24 h (for aerobic microorganisms) and 37 °C for 48 h (for *C. perfringens* and *C. sporogenes*) in an anaerobic chamber (Whitley DG250 Anaerobic Workstation). Yeasts were grown in Yeast Extract Peptone Dextrose agar (YPDa, Duchefa, Biochemie, Haarlem, The Netherlands) at 25 °C for 48 h.

### 4.2. Disc Diffusion Assay (DDA)

Each inoculum was prepared by resuspending isolated colonies of each strain, previously grown on TSAYE or YPD agar, in sterile ¼ Ringer’s solution (Biokar Diagnostics) to obtain turbidity equivalent to 0.5 on the McFarland scale (BioMérieux, Marcy-l’Etoile, France). The antimicrobial activity of EOs was screened by disc diffusion assay (DDA) according to the methodology described by Carvalho et al. [23] and Zaika [43], with some modifications. Briefly, Petri dishes prepared with Mueller–Hinton agar (MHA; Biokar, France) or YPD agar for yeasts were dried and uniformly spread with the standardized inoculum using a swab. Emulsified EOs were prepared with and without polysorbate 80 food-grade solution (Sigma). Falcon tubes of 50 mL (Sarstedt, Germany) containing only Muller–Hinton broth (33.4% *v*/*v*) (MHb—Biokar, France) or MHb and polysorbate 80 (10% *v*/*v*) were preheated at 50 °C for 10 min at 500 rpm in an orbital incubator (Orbital Labwit ZWYR—200D, Frilabo, Maia, Portugal). EOs were then added in each Falcon tube to obtain a final concentration of 66.6% (*v*/*v*) each and heated at 50 °C. Filter paper discs (Whatman No. 5, 6 mm diameter, Oxoid, Hampshire, UK) were impregnated with each EOs solution for 15 min. The discs were left at room temperature for 30 min until evaporation was complete. The loaded discs were placed on the surface of the inoculated agar. Commercial antibiotic discs, imipenem monohydrates (5 mg/mL, Sigma), chloramphenicol (50 mg/mL, Sigma) and amphotericin B (250 µg/mL, Sigma), were used as positive controls. Discs containing only polysorbate 80 were used as negative controls. Plates were incubated at 37 °C for 24 h, 37 °C for 48 h, and 25 °C for 48 h for aerobic bacteria, anaerobic bacteria, and yeasts, respectively. Antimicrobial activity was evaluated by measuring the total diameter (in millimeters) of the inhibition zone, which included both the disc and the surrounding clear halo. Inhibition was only considered if the halos were greater than 10 mm [34]. The DDA assay was performed in duplicate.

### 4.3. Minimum Inhibitory Concentration (MIC)

The minimum inhibitory concentrations (MIC) were determined using a modified version of the procedures described by Aumeeruddy-Elalfi et al. [37], using 96-well microtitre plates. From the EOs solutions prepared as described above, successive dilutions (1:1) were made in MHb and MHb with polysorbate (10% *v*/*v*) and Yeast Extract Peptone dextrose broth (YPDb—VWR, EUA) and YPDb with polysorbate for yeasts (first well with a concentration of 50% *v*/*v*). One hundred and fifty microliters of EOs solutions were added to each well of the microtitre plate. Then, 50 µL of each microorganism suspension (prepared as described above) was added to each well of the microtitre plates. A negative control without inoculation was included, and antibiotics were used as positive controls: imipenem (10 µg, Oxoid), chloramphenicol (30 µg, Oxoid), and amphotericin B (250 µg/mL, Sigma). To assess the minimum microbicidal concentration (MMC), 3 μL from each well showing no visible microbial growth was plated on MHa and incubated under the conditions described above. Controls were performed with polysorbate and MHb with polysorbate (10% *v*/*v*). MICs were carried out in three independent trials, each in duplicate.

### 4.4. Statistical Analysis

The statistical analysis was conducted using Microsoft^®^ Excel^®^ para Microsoft 365 MSO (versão 2411, Microsoft, Washington, DC, USA). The Student’s *t*-test was applied to determine the significance of differences between the means of two groups. The built-in Excel function T.TEST was utilized, with a significance level set at *p* < 0.05. Before conducting the test, the data were reviewed to ensure proper formatting and consistency. This method provided an accessible and reliable way to evaluate differences between the groups.

## 5. Conclusions

Based on the results obtained, it can be concluded that the methodology used to evaluate antimicrobial activity and determine the MICs of the essential oils (EOs) studied indicates that the use of polysorbate 80 reduces the efficacy of these oils. Specifically, the antimicrobial activity was reduced when the EOs were emulsified with polysorbate 80 compared with their application without this surfactant. This finding suggests that the presence of polysorbate 80 may interfere with the antimicrobial properties of the EOs, possibly by altering their chemical interactions or bioavailability. However, it is important to note that no published research directly compares the antimicrobial efficacy of EOs with and without polysorbate 80. Therefore, while our findings suggest a potential negative effect of polysorbate 80 on the performance of EOs, further research is needed to establish a definitive relationship. In addition, the variability in the methods used in different studies may contribute to discrepancies in the results obtained. This variability, which includes differences in experimental conditions, types of microorganisms tested, and methods of application of the EOs, may significantly impact the efficacy of EOs in practical contexts. Therefore, it is crucial to consider these factors when interpreting the results of EOs and their antimicrobial applications.

Future research should focus on investigating the mechanisms by which polysorbate 80 affects the antimicrobial activity of EOs. In addition, studies comparing the effects of different surfactants and their concentrations on the efficacy of EOs could provide valuable insights for optimizing the use of these oils in antimicrobial applications, such as the food industry (acting as potential natural preservatives) and pharmaceutical and cosmetic industries (e.g., replacing antibiotics and other antimicrobial compounds).

## Figures and Tables

**Table 1:** Zones of growth inhibition (mm; mean ± standard deviation) showing antimicrobial activity (including the disc diameter of 6.0 mm) of oregano and thyme EOs with and without polysorbate against different microorganisms.

DDA		With Polysorbate		Without Polysorbate	
		Oregano	Thyme	Oregano	Thyme
Gram-positive	*Bacillus cereus* ESB014	37.7 ± 0.5	28.4 ± 2.8	39.7 ± 3.7	36.1 ± 2.2
	*Bacillus subtilis* ESB015	22.9 ± 0.4	18.6 ± 0.5	24.4 ± 2.3	23.2 ± 1.1
	*Bacillus stearothermophilus* ESB016	34.2 ± 0.5	26.6 ± 1.5	35.1 ± 2.9	41.0 ± 4.9
	*Clostridium perfringens* 1.16	20.7 ± 3.9	20.9 ± 2.1	12.1 ± 1.0	22.0 ± 1.0
	*C. perfringens* 1.19	18.9 ± 1.6	20.4 ± 1.1	37.4 ± 0.0	16.1 ± 2.1
	*Clostridium sporogenes* 1.31	20.9 ± 0.1	25.7 ± 0.7	39.7 ± 1.1	13.5 ± 0.9
	*C. sporogenes* 1.34	17.3 ± 2.6	19.0 ± 0.8	37.4 ± 0.0	18.8 ± 1.7
	*C. sporogenes* 1.61	25.8 ± 0.1	28.1 ± 0.4	11.1 ± 0.4	14.1 ± 1.8
	*Enterococcus casseliflavus* DSMZ 20680	27.9 ± 1.1	17.3 ± 2.1	28.0 ± 2.5	26.8 ± 1.1
	*Enterococcus faecalis* ATCC 29212	21.7 ± 5.2	10.8 ± 0.2	29.6 ± 3.5	28.9 ± 1.1
	*E. faecalis* DSMZ 12956	23.1 ± 2.1	10.1 ± 0.5	36.6 ± 2.3	16.8 ± 2.0
	*Enterococcus faecium* DSMZ 13590	36.3 ± 0.5	16.7 ± 3.2	34.5 ± 2.3	28.3 ± 4.4
	*Enterococcus flavescens* DSMZ 7370	40.2 ± 3.6	37.4 ± 1.1	37.2 ± 0.8	30.1 ± 0.4
	*Enterococcus gallinarum* DSMZ 20628	30.4 ± 0.0	26.3 ± 0.4	29.6 ± 2.4	28.7 ± 3.2
	*Listeria innocua* 2030c	34.4 ± 0.8	29.4 ± 2.5	39.5 ± 3.3	32.1 ± 1.7
	*Listeria monocytogenes* 7946	31.1 ± 0.2	31.1 ± 0.8	38.4 ± 2.5	36.0 ± 1.4
	*L. monocytogenes* 7947	31.5 ± 3.3	32.1 ± 2.4	35.9 ± 2.4	33.7 ± 5.9
	*L. monocytogenes* SCOTT A	14.0 ± 2.4	11.4 ± 1.7	14.8 ± 0.8	13.0 ± 2.8
	*Staphylococcus aureus* ATCC 29213	30.8 ± 1.1	28.4 ± 3.6	28.8 ± 1.3	29.9 ± 2.5
	*S. aureus* 18N (Methicillin-resistant *S. aureus*—MRSA)	35.1 ± 3.2	30.5 ± 0.4	30.0 ± 1.6	34.7 ± 3.0
	*S. aureus* 2037 M1 (Methicillin-sensitive *S. aureus*—MSSA)	30.2 ± 0.5	29.3 ± 2.6	31.0 ± 0.1	30.0 ± 1.6
Gram-negative	*Acinetobacter baumannii* (resistant) ESB028	36.5 ± 0.1	30.1 ± 3.5	37.4 ± 4.0	32.6 ± 3.7
	*A. baumannii* (sensitive-1) ESB029	36.8 ± 0.0	28.1 ± 2.7	37.7 ± 3.5	38.3 ± 3.3
	*A. baumannii* (sensitive-2) ESB032	35.8 ± 0.8	30.1 ± 0.6	33.5 ± 0.0	32.4 ± 0.0
	*Acinetobacter calcoaceticus* (resistant) ESB030	29.8 ± 1.8	26.0 ± 1.3	29.4 ± 1.5	30.4 ± 1.2
	*A. calcoaceticus* (sensitive) ESB031	32.5 ± 1.8	30.1 ± 0.8	33.0 ± 1.8	31.4 ± 1.4
	*Escherichia coli* ATCC 25922	33.0 ± 1.9	24.7 ± 0.5	33.9 ± 1.8	31.9 ± 2.7
	*Klebsiella pneumoniae* ESB011	16.6 ± 2.8	16.8 ± 3.3	24.7 ± 2.1	20.4 ± 3.5
	*Proteus mirabilis* ESB027	32.4 ± 2.7	28.6 ± 0.4	30.1 ± 2.8	36.7 ± 1.8
	*Proteus vulgaris* ESB012	23.6 ± 1.0	16.0 ± 1.8	24.9 ± 2.1	22.6 ± 2.8
	*Pseudomonas aeruginosa* ESB048	22.2 ± 0.7	17.8 ± 1.6	21.1 ± 2.1	18.5 ± 0.2
	*Salmonella* Braenderup ESB007	23.3 ± 1.8	16.8 ± 0.4	25.1 ± 1.6	22.1 ± 2.5
	*Salmonella* Enteritidis ESB008	23.4 ± 0.0	18.8 ± 2.1	23.3 ± 0.5	20.9 ± 1.5
	*Salmonella* Enteritidis 417536	17.0 ± 0.7	16.1 ± 1.3	19.6 ± 1.7	20.5 ± 2.5
	*Salmonella* Enteritidis 545047	16.3 ± 3.0	18.4 ± 3.4	19.8 ± 0.3	20.0 ± 0.2
	*Salmonella* Tiphymurium ESB009	30.7 ± 1.3	24.6 ± 0.4	33.3 ± 3.0	29.9 ± 1.8
	*Yersinia enterocolitica* ESB024	36.1 ± 3.2	31.2 ± 1.3	40.4 ± 4.0	47.8 ± 3.3
	*Y. enterocolitica* NCTC10406	22.1 ± 1.1	18.6 ± 1.5	23.3 ± 2.5	21.5 ± 1.0
Yeasts	*Candida albicans* ESB025	44.5 ± 0.4	35.7 ± 0.4	55.3 ± 2.6	50.7 ± 2.8
	*Saccharomyces cerevisiae* ESB026	44.5 ± 4.5	26.7 ± 4.4	50.0 ± 1.8	37.5 ± 0.1

**Table 2:** Minimum Inhibitory Concentration (MIC) (results are expressed in % of EO) of oregano and thyme EOs with and without polysorbate against different microorganisms.

	MICs	With Polysorbate		Without Polysorbate	
		Oregano	Thyme	Oregano	Thyme
Gram-positive	*B. cereus* ESB014	12.50	3.12	0.02	0.19
	*B. subtilis* ESB015	12.50	6.25	0.02	0.09
	*B. stearothermophilus* ESB016	12.50	6.25	0.39	0.39
	*C. perfringens* 1.16	3.12	3.12	0.02	0.09
	*C. perfringens* 1.19	3.12	6.25	0.02	0.09
	*C. sporogenes* 1.31	6.25	6.25	0.02	0.19
	*C. sporogenes* 1.34	6.25	12.50	0.02	0.19
	*C. sporogenes* 1.61	3.12	6.25	0.02	0.19
	*E. casseliflavus* DSMZ 20680	6.25	6.25	0.09	0.09
	*E. faecalis* ATCC 29212	12.50	12.5	0.02	0.19
	*E. faecalis* DSMZ 12956	12.50	6.25	0.02	0.19
	*E. faecium* DSMZ 13590	6.25	6.25	0.02	0.19
	*E. flavescens* DSMZ 7370	6.25	6.25	0.09	0.02
	*E. gallinarum* DSMZ 20628	6.25	6.25	0.02	0.19
	*L. innocua* 2030c	3.12	6.25	0.02	0.09
	*L. monocytogenes* 7946	0.39	3.12	0.02	0.05
	*L. monocytogenes* 7947	0.39	0.78	0.02	0.05
	*L. monocytogenes* SCOTT A	3.12	6.25	0.02	0.09
	*S. aureus* ATCC 29213	3.12	6.25	0.02	0.05
	*S. aureus* 18N (MRSA)	6.25	6.25	0.02	0.09
	*S. aureus* 2037 M1 (MSSA)	0.78	3.12	0.02	0.09
Gram-negative	*A. baumannii* (resistant) ESB028	1.56	1.56	0.02	0.05
	*A. baumannii* (sensitive-1) ESB029	1.56	1.56	0.02	0.05
	*A. baumannii* (sensitive-2) ESB032	1.56	1.56	0.02	0.05
	*A. calcoaceticus* (resistant) ESB030	0.78	1.56	0.02	0.05
	*A. calcoaceticus* (sensitive) ESB031	0.78	1.56	0.02	0.05
	*E. coli* ATCC 25922	3.12	3.12	0.09	0.19
	*K. pneumoniae* ESB011	6.25	3.12	0.09	0.19
	*P. mirabilis* ESB027	1.56	3.12	0.02	0.05
	*P. vulgaris* ESB012	3.12	12.50	0.09	0.19
	*P. aeruginosa* ESB048	6.25	12.50	0.05	0.05
	*Salmonella* Braenderup ESB007	3.12	12.50	0.09	0.09
	*Salmonella* Enteritidis ESB008	3.12	12.50	0.09	0.09
	*Salmonella* Enteritidis 417536	1.56	3.12	0.02	0.05
	*Salmonella* Enteritidis 545047	3.12	6.25	0.02	0.05
	*Salmonella* Tiphymurium ESB009	1.56	3.12	0.09	0.09
	*Y. enterocolitica* ESB024	1.56	3.12	0.02	0.09
	*Y. enterocolitica* NCTC10406	1.56	3.12	0.02	0.09
Yeasts	*C. albicans* ESB025	3.12	0.78	0.02	0.09
	*S. cerevisiae* ESB026	0.78	1.56	0.02	0.02

**Table 3:** The microbial strains investigated in this study and their respective origin.

Microorganisms	Species	Source
Gram-positive	*B. cereus* ESB014	ESB culture collection
	*B. subtilis* ESB015	
	*B. stearothermophilus* ESB016	
	*Listeria monocytogenes* SCOTT A	
	*Listeria innocua* 2030c	
	*S. aureus* 18N (MRSA)	
	*S. aureus* 2037 M1 (MSSA)	
	*C. sporogenes* 1.31	
	*C. sporogenes* 1.34	
	*C. sporogenes* 1.61	
	*C. perfringens* 1.16	
	*C. perfringens* 1.19	
	*E. faecalis* ATCC 29212	ATCC
	*S. aureus* ATCC 29213	
	*E. faecalis* DSMZ 12956	DSMZ
	*E. faecium* DSMZ 13590	
	*E. flavescens* DSMZ 7370	
	*E. casseliflavus* DSMZ 20680	
	*E. gallinarum* DSMZ 20628	
	*L. monocytogenes* L7946	[42]
	*L. monocytogenes* L7947	
Gram-negative	*A. baumannii* R ESB028	ESB culture collection
	*A. baumannii* S-1 ESB029	
	*A. baumannii* S-2 ESB032	
	*A. calcoaceticus* R ESB030	
	*A. calcoaceticus* S ESB031	
	*K. pneumoniae* ESB011	
	*P. mirabilis* ESB027	
	*P. vulgaris* ESB012	
	*P. aeruginosa* ESB048	
	*S.* Braenderup ESB007	
	*S.* Enteritidis ESB008	
	*S.* Enteritidis 417536	
	*S.* Enteritidis 545047	
	*S.* Typhimurium ESB009	
	*Y. enterocolitica* ESB024	
	*E. coli* ATCC 25922	ATCC
	*Y. enterocolitica* NCTC 10406	NCTC
Yeasts	*C. albicans* ESB025	ESB
	*S. cerevisiae* ESB026	

## Data Availability

The original contributions presented in this study are included in the article. Further inquiries can be directed to the corresponding author.
